# Racial Disparities in Quantitative MRI for African American and White Men with Prostate Cancer

**DOI:** 10.21203/rs.3.rs-2547854/v1

**Published:** 2023-02-15

**Authors:** Fatemeh Zabihollahy, Qi Miao, Ida Sonni, Sitaram Vangala, Harrison Kim, William Hsu, Anthony Sisk, Robert Reiter, Steven Raman, Kyunghyun Sung

**Affiliations:** 1 Department of Radiological Sciences, David Geffen School of Medicine, University of California, Los Angeles, CA, USA; 2 Department of Radiology, The First Affiliated Hospital of China Medical University, Shenyang City, Liaoning Province, China; 3 Department of Medicine Statistics Core, David Geffen School of Medicine, University of California, Los Angeles, CA, USA; 4 Department of Radiology, University of Alabama at Birmingham, Birmingham, AL, USA; 5 Department of Pathology, David Geffen School of Medicine, University of California, Los Angeles, CA, USA; 6 Department of Urology, David Geffen School of Medicine, University of California, Los Angeles, CA, USA

## Abstract

The risk of prostate cancer (PCa) is strongly influenced by race and ethnicity. The purpose of this study is to investigate differences in the diagnostic performance of multiparametric MRI (mpMRI) in African American (AA) and white (W) men. 111 patients (37 AA and 74 W men) were selected from the study’s initial cohort of 885 patients after matching age, prostate-specific antigen, and prostate volume. The diagnostic performance of mpMRI was assessed using detection rates (DRs) and positive predictive values (PPVs) with/without combining *K*^*trans*^ (volume transfer constant) stratified by prostate zones for AA and W sub-cohorts. The DRs of mpMRI for clinically significant PCa (csPCa) lesions in AA and W sub-cohort with PI-RADS scores ≥ 3 were 67.3% *vs*. 80.3% in the transition zone (TZ; *p*=0.026) and 81.2% *vs*. 76.1% in the peripheral zone (PZ; *p*>0.9). The *K*^*trans*^ of csPCa in AA men was significantly higher than in W men (0.23±0.08 min^−1^ *vs*. 0.19±0.07 min^−1^; *p*=0.022). This emphasizes that there are race-related differences in the performance of mpMRI and quantitative MRI measures that are not reflected in age, PSA, and prostate volume.

## INTRODUCTION

The risk of prostate cancer (PCa) is strongly influenced by race and ethnicity.^1^ In particular, African American (AA) men in the general US population have a higher likelihood of PCa-related death than White (W) men due to increased incidence and poorer survival after diagnosis.^2,3^ Multiple studies have suggested that socioeconomic factors and healthcare access may account for the difference.^2,4,5,6^ However, a growing body of literature also shows that genetic and biological factors may be equally implicated in developing these discrepancies.^7,8^ The underlying causes are complex and likely multifactorial.^9^ Therefore, understanding the impact of biological heterogeneity in patients from different racial/ethnic backgrounds is crucial for reducing the observed gaps in clinical outcomes.

Multiparametric MRI (mpMRI) allows for the exploration of the biological and molecular characteristics of PCa with a combination of anatomic and functional information. Dynamic contrast-enhanced MRI (DCE-MRI), as part of mpMRI, measures microvascular perfusion by monitoring the dynamic change of MRI contrast agent in the target tissue.^10,11^ Increased perfusion is associated with a higher grade of PCa requiring more aggressive management. Differences in quantitative DCE-MRI (qDCE) parameters can potentially explain the biological differences noted among AA and W men and ultimately improve the characterization of clinically significant PCa (csPCa) in patients with different ethnic backgrounds when correctly accounted for the interpretation of mpMRI.^12,13^ Also, several studies have shown that AA men present with higher prostate volume (PV) and prostate-specific antigen (PSA) than W men.^9,14^ Therefore, these clinical variables need to be adjusted to minimize the bias when investigating potential imaging differences between AA and W men.

This study aims to first investigate the diagnostic performance of mpMRI in AA and W populations after matching clinical variables, such as patient age, PSA, and PV. Radiology-pathology correlations were analyzed in AA and W sub-cohorts who underwent 3T mpMRI prior to radical prostatectomy to estimate cancer prevalence (CP), detection rate (DR), and positive predictive value (PPV) stratified by prostate zones and to explore differences between the two race groups. Moreover, we assessed whether any differences exist in quantitative MRI parameters of the pathology- and MRI-based lesions between AA and W sub-cohorts. Finally, we tested the feasibility of adding the quantitative MRI parameters to the Prostate Imaging Reporting and Data System (PI-RADS) for improved diagnosis of PCa in both AA and W sub-cohorts.

## MATERIAL AND METHODS

### Study population

**Figure 1:**
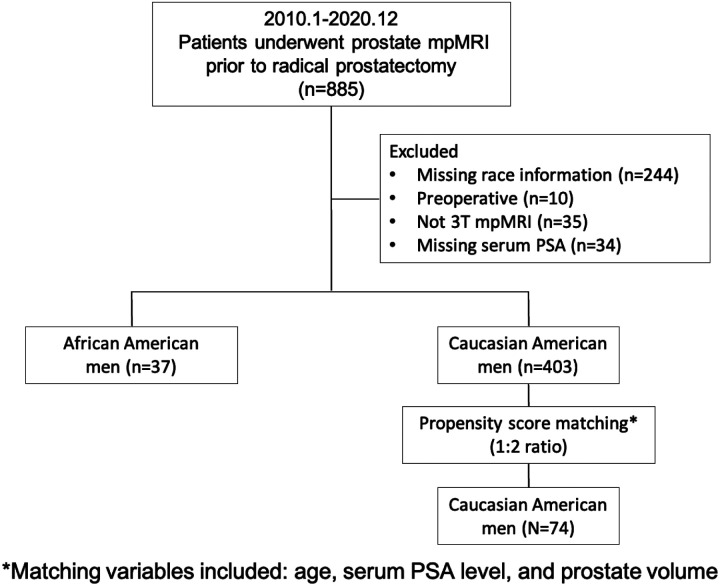
The inclusion workflow of the study population.

This single institutional retrospective study was approved by the UCLA institutional review board (IRB) with a waiver of the requirement for informed consent and was conducted in compliance with the United States Health Insurance Portability and Accountability Act (HIPAA) of 1996. The initial study cohort comprised 885 consecutive patients who underwent mpMRI prior to prostatectomy from July 2010 to December 2020. We excluded all patients meeting one or more of the following criteria: 1) unknown/missing race information; 2) prior treatment for PCa (e.g., radiation therapy, focal ablation, androgen deprivation therapy); 3) missing mpMRI in 3T scanners; 4) missing preoperative serum prostate-specific antigen (PSA) measurement. After reviewing the electronic medical record, patient age, self-identified race/ethnicity, clinical (serum PSA levels prior to surgery), imaging (mpMRI), and pathology reports were recorded. We applied a propensity score caliper matching algorithm to match AA to W men in a 1:2 ratio with the variables, including patient age, PSA, and PV. These clinical variables are known to be associated with the risk factors for PCa diagnosis, and studies have shown that AA men present with higher PSA values when compared with W men.^9,14^ The differences in age, PSA, PSAD, and PV between AA and W populations before propensity score matching are shown in Table 1. The sample size of the AA and W groups was unbalanced, and a significant difference in PV (p<0.05) existed, which can be a confounding factor. We utilized propensity score matching to minimize the bias due to the confounding variables when comparing AA and W men, similar to previous studies.^15,16,17,18^ We adjusted these covariates, including age, PSA, and PV, to balance them within the strata of the propensity score between the two groups. A total of 111 AA and W men were included in the final quantitative analysis (Figure 1).

### MRI acquisition and analysis

The mpMRI was performed on one of the 3T scanners (Magnetom, Prisma, Skyra, Vida, and Verio; Siemens Healthineers, Erlangen, Germany) using standardized protocols. All preoperative mpMRIs were interpreted by an abdominal imaging fellow and then reviewed by one of three board-certified attending abdominal radiologists with 5–20 years of experience as part of the standard of care in our institute. All mpMRI scans were interpreted using the Prostate Imaging Reporting and Data System (PI-RADS) v2.1 guidelines.

Apparent diffusion coefficient (ADC) maps were generated via the in-line postprocessing (Siemens Healthineers, Erlangen, Germany) using the least-squares curve fitting method when the DWI images were obtained with four *b* values (0/100/400/800). The DCE-MRI was implemented with an ultrafast 3D spoiled gradient-echo sequence without fat saturation. Five images were acquired before injecting gadopentetate dimeglumine (Magnevist; Bayer, Wayne, NJ) at a dose of 0.1 mmol/kg through a peripheral vein at a rate of 2 mL/sec via a mechanical injector, and approximately 70 images were acquired after that without delay between the acquisitions with a temporal resolution of 5–6 seconds. The number of image slices was 20, and the slice thickness was 3.6 mm. The detailed MRI sequence parameters are shown in Table 2.

For quantitative analysis of DCE-MRI, we used the standard Tofts model defined as:

$$C_{t}\left(t\right)=K^{\text{trans}}\int_{0}^{t}C_{p}\left(\tau \right)e^{-k_{ep}\left(t-\tau \right)}d\tau ,$$

where *C*_*t*_(*t*) is the total tissue contrast agent concentration, *C*_*p*_(*t*) is the time-varying blood plasma concentration after a bolus of gadolinium is administered, *K*^*trans*^ is the volume transfer constant (wash-in rate; min^−1^), and *k*_*ep*_ is the blood influx rate (wash-out rate; min^−1^). Among multiple arterial input function (AIF) options, a population-averaged Parker AIF was used to obtain *K*^*trans*^ and *k*_*ep*_ using DCE-MRI images.^19,20^ The qDCE analysis was implemented, blinded to race/ethnicity, using a lab-made software package with MATLAB (MathWorks, Natick, MA), compliant with the Quantitative Imaging Biomarkers Alliance (QIBA) DCE-MRI quantitation profile.^21^

### Radiology-pathology correlation

Genitourinary pathology technicians prepared thin-section whole-mount histopathology (WMHP) slices; each prostate gland was in the axial plane, perpendicular to the urethra from anterior to posterior from the apex to the base in 5-mm increments using a 3D printed mold derived from the preoperative MRI. Each whole mount slice was fixed for 24 hours and embedded in paraffin. After hematoxylin and eosin (H&E) staining, each whole-mount slice was digitally photographed. Two genitourinary pathologists manually delineated the PCa tumor boundary on each image slice and assigned a Gleason score (GS) and the International Society of Urological Pathology (ISUP) grade for each PCa lesion.^22^ The lesions with ISUP grade 2 or higher (GS ≥ 3+4) were defined as csPCa. For MRI-positive and/or pathology-positive lesions, both an MRI scientist (K.S. with 15+ years of experience in analyzing MRI data) and an abdominal radiologist (Q.M. with 5+ years of experience in prostate mpMRI interpretation) retrospectively reviewed all cases and manually annotated regions-of-interest (ROIs) encompassing the entire lesion on slices of both the ADC and *K*^*trans*^ maps.

**Figure 2:**
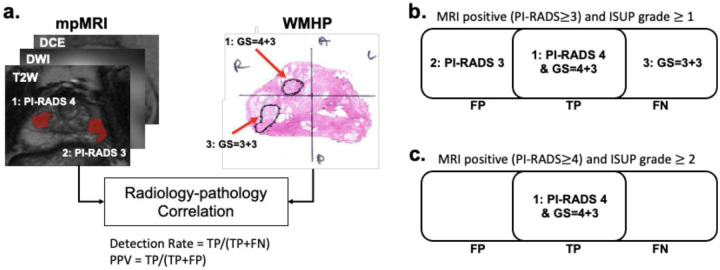
Radiology-pathology correlation using (a) mpMRI and WMHP, and illustration of true positive (TP), false positive (FP), and false negative (FN) lesions with different MRI/histopathological finding groups: (b) ISUP grade ≥ 1 with PI-RADS ≥ 3 (detection rate = 50% and PPV = 50%) or (c) ISUP grade ≥ 2 with PI-RADS ≥ 4 (detection rate = 100% and PPV = 100%).

An MRI-positive and pathology-positive lesion was labeled as true positive (TP). An MRI-positive but pathology-negative lesion was labeled as false positive (FP), while an MRI-negative but pathology-positive lesion was labeled as false negative (FN). Figure 2 contains an example of our radiology-pathology correlation analysis with mpMRI and WMHP and the procedure of labeling lesions as TP, FP, and FN when different criteria are applied. As seen in Figure 1a, there are two MRI lesions with PI-RADS 4 and 3 and two pathology lesions with ISUP grades of 3 and 1. Comparing lesions on MRI and WMHP reveals that lesion #1 appeared on both MRI and WMHP. In contrast, lesion #2 was only shown on MRI, and lesion #3 was only localized on WMHP. We showed examples of the different definitions of TP/FP/FN when we defined MRI positives as PI-RADS ≥ 3 and pathology positives as ISUP grade ≥ 1 (Figure 1b) and when we defined MRI positives as PI-RADS ≥ 4 and pathology positives as ISUP grade ≥ 2 (Figure 1c). The final number of PCa lesions represents the sum of all lesions identified by WMHP (i.e., TP+FN).

We assessed the diagnostic performance of mpMRI between matched W and AA men by calculating detection rates, DR=TP/(TP+FN), and positive predictive values, PPV=TP/(TP+FP). The location of each lesion (TP, FP, and FN) was recorded on the sector map described by PI-RADS v2.1, and the number of each TP, FN and FP lesion was normalized by the number of sectors where the lesion was distributed on. Collecting the per-lesion-based location information yielded the weighted sum of TP, FP, and FN lesions in each sector, which was used to obtain the per-lesion diagnostic performance of mpMRI (DR and PPV) in both AA and W men.

**Figure 3:**
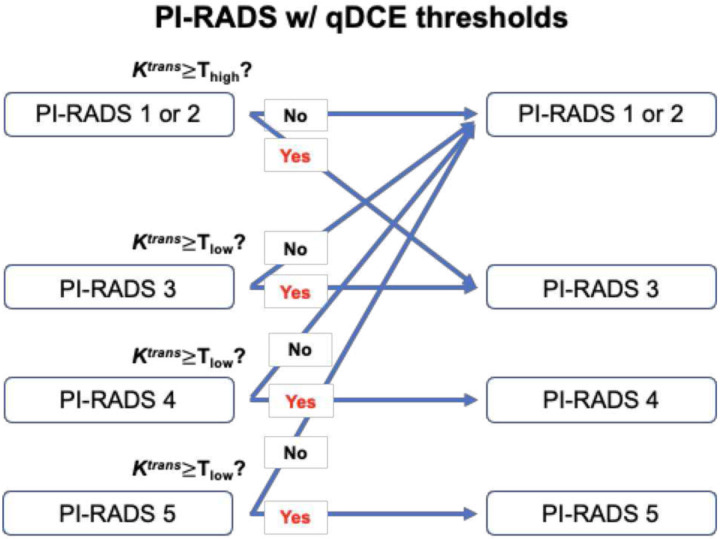
Updated PI-RADS scoring with two qDCE thresholds (T_high_ and T_low_). PI-RADS 1 or 2 score is progressively upgraded to PI-RADS 3 when K^trans^ is higher than the high threshold (T_high_), and PI-RADS 3–5 scores are downgraded to PI-RADS 1 or 2 when K^trans^ is lower than the low threshold (T_low_).

**Figure 4:**
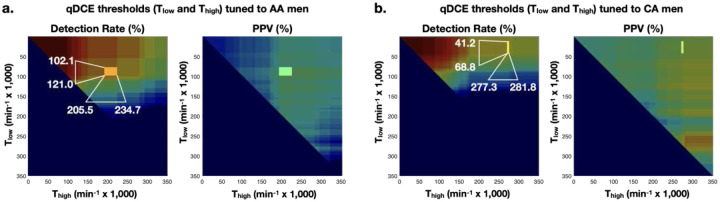
Estimation of the qDCE thresholds (T_low_ and T_high_) in DRs and PPVs for (a) African American and (b) White men. A brute-force search method was used with different combinations of T_low_ and T_high_, ranging from 0 to 0.35 min^−1^., and the qDCE thresholds are highlighted when the diagnostic performance is optimal, as the highest DR without a decrease in PPV, for (a) AA and (b) W men.

Figure 3 illustrates a simple approach to updating the PI-RADS score by adding qDCE (*K*^*trans*^) thresholds. PI-RADS 1 or 2 were upgraded to PI-RADS 3 if *K*^*trans*^ was higher than the upper threshold (T_high_). In contrast, PI-RADS 3–5 were downgraded to PI-RADS 1 or 2 if *K*^*trans*^ was lower than the lower threshold (T_low_). The thresholds were identified using a simple brute-force search algorithm to find *K*^*trans*^ values that increased DRs without compromising PPVs, as indicated by the arrows in Figure 4.

Table 3 indicates the results for whole PCa lesions. While the DR of total PCa lesions in W men was significantly higher than that in AA men, no significant difference was witnessed for PPVs.

### Statistical analysis

All data were statistically analyzed using SPSS v26.0 (IBM Corp, Armonk, NY, USA). Baseline patient demographics between AA and W men were compared using the Mann-Whitney U test for continuous variables. The weighted Pearson’s chi-square test was used to analyze the categorical variables including DR and PPV in which the weighted sum of each variable was adjusted by the number of observations. A P-value less than 0.05 was considered statistically significant.

## RESULTS

Table 4 shows the patient and lesion characteristics after matching the propensity score with the variables of patient age, PSA, and prostate volume. Among the total of 174 PCa lesions on WMHP, 63 and 111 lesions were identified for AA and W sub-cohorts, respectively. The average number of PCa lesions per patient was 1.6. The average number of pathology-based lesions per patient for AA men was 1.7, which did not significantly differ from that for W men (1.5). Similarly, among the total of 126 MRI-positive lesions, the average number of MRI-based lesions per patient for AA men was 1.2, which did not significantly differ from that for W men (1.1).

**Figure 5:**
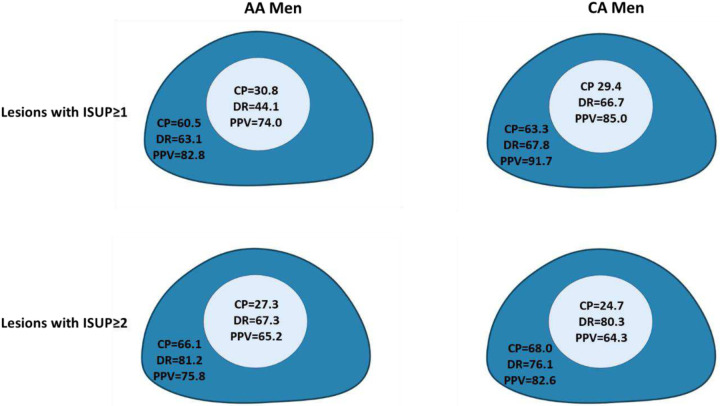
Diagnostic performance of mpMRI in terms of cancer prevalence (CP), detection rate (DR), and positive predictive value (PPV) from top to bottom for lesions with ISUP ≥ 1 and 2 for African American and White men. All numbers are in percent.

Detailed diagnostic performances of mpMRI between AA and W sub-cohorts are compared in Table 5 for csPCa lesions. The difference between the DRs in AA and W sub-cohorts was not significant except for the transition zone (TZ) (67.3% vs. 80.3%, *p*=0.026). However, the opposite pattern was witnessed for the PPVs of csPCa; the difference between AA and W sub-cohorts in the peripheral zone (PZ) or the whole prostate gland was significant, but the difference in the TZ was not (65.2% vs. 64.3%, *p*=0.06). Figure 5 illustrates the diagnostic performance of mpMRI for the entire PCa lesions (or csPCa lesions) in AA and W sub-cohorts by prostate zones, presenting substantial differences between the mpMRI performance in terms of CP, DR, and PPV for cohorts in this study. Table 5 indicates the results for all PCa lesions.

**Figure 6:**
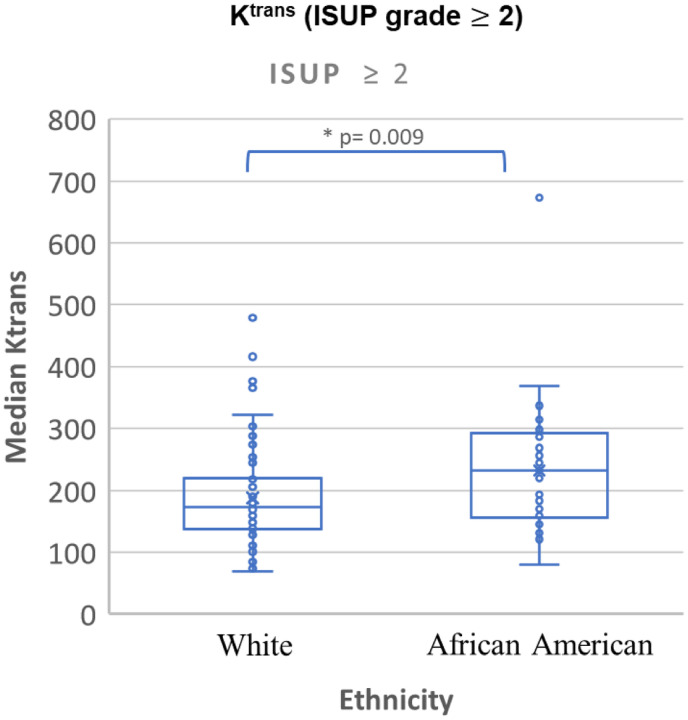
Quantitative DCE for csPCa lesions in African American and White men.

Quantitative DCE-MRI and ADC parameters in AA and W sub-cohorts are summarized in Table 6. Notable differences between the two cohorts were observed in the *K*^*trans*^ of tumors with ISUP grades 2 and 3 (*p*=0.022 and 0.050, respectively) and for tumors with ISUP ≥ 1 and 2 (*p*=0.033 and 0.009, respectively). Additionally, the tumors of AA men had significantly higher *K*^*trans*^ than the W sub-cohort when the PI-RADS score was ≥ 3 (*p*=0.013). However, the *k*_*ep*_ and ADC were not significantly different between the two races. Figure 6 shows the boxplots of *K*^*trans*^ of AA and W sub-cohort.

Table 7 compares the diagnostic performance of mpMRI between AA and W sub-cohorts when PI-RADS was updated using *K*^*trans*^ only for the AA sub-cohort (first two columns) or both AA and W sub-cohorts (second two columns). The *K*^*trans*^ thresholds were T_low_ = 102 and T_high_ = 206 min^−1^ × 10^−3^ for AA men while those for W men were T_low_ = 41 and T_high_ = 277 min^−1^ × 10^−3^. Figure 4 illustrates the identification of the *K*^*trans*^ thresholds with the variation of DRs and PPVs according to T_low_ and T_high_.

Our findings revealed that updating PI-RADS using *K*^*trans*^ for AA sub-cohort increased the DRs of csPCa by 10%, 9%, and 11% in the whole prostate gland, TZ, and PZ, respectively. When PI-RADS was updated using *K*^*trans*^ for the AA sub-cohort, the DR of csPCa in the TZ of AA men was not significantly different from that of W men (76.2% *vs*. 80.3%, *p*=0.180).

When the *K*^*trans*^ was added to PI-RADS for AA and W sub-cohorts, both DR and PPV of total PCa lesions (ISUP ≥ 1) were increased (+11.1% in DR and +4.2% in PPV for AA men and +12.6% in DR and +2.5% in PPV for W men). Similarly, the diagnostic performance of total PCa lesions (DR and PPV) in both TZ and PZ was ameliorated for both AA and W groups when *K*^*trans*^ was added to PI-RADS.

## DISCUSSION

AA men present with more advanced PCa and an age-adjusted PCa mortality rate than W men.^23^ A recent investigation including 600 patients showed that PCa detection using PI-RADS was not statistically different between AA and W sub-cohort.^24^ Still, another study (n=194) identified that AA men were at a significantly higher risk of having csPCa when mpMRI is negative (PI-RADS 1 or 2).^25^ These results indicate that the current PI-RADS-based interpretation may not be sensitive enough to account for underlying ethnic/race-specific biological differences in PCa. In this study, we found that one of the qDCE parameters, *K*^*trans*^, was significantly higher in AA men than one in W men with csPCa matching for ages, serum PSA levels, and prostate volumes. Thus, accounting for qDCE in the analysis of mpMRI can improve the understanding and characteristics of csPCa between AA and W sub-cohorts and may further enhance the diagnostic performance of PCa in AA sub-cohorts.

All quantitative MRI measures (*K*^*trans*^, *k*_*ep*,_ and ADC) were calculated by averaging values within volumetric ROIs encompassing the entire lesion on multiple slices. The volumetric average would reduce dependencies on potential variations of the lesion annotations. Moreover, similar trends in quantitative MRI measures were observed in median values within the ROIs, which further assures the reproducibility of the image analysis.

The racial difference in *K*^*trans*^ was statistically significant in the PCa of ISUP 2 or 3, but not in ISUP 1 (*p*=0.664). Our findings suggested that the highest contributor to the difference in *K*^*trans*^ between AA and W sub-cohorts was in the ISUP 2 PCa. Furthermore, when categorizing lesions based on PI-RADS v2.1 scores, the difference in *K*^*trans*^ was noted in the lesions with PI-RADS ≥ 3, but not in PI-RADS 3, confirming that the PI-RADS 3 classification remains uncertain to determine the PCa malignancy.

The distinct characteristics of *K*^*trans*^ between AA and W men could reflect biological tumor differences among men from different racial backgrounds, as suggested by several studies investigating genetic/epigenetic factors and the influence of the tumor microenvironment.^7,8,26^ If present, any race-based parameters that reflect differences in tumor biology should be used to improve diagnosis in AA men, especially when interpreting mpMRI. Our study showed the feasibility of utilizing race-specific thresholds of qDCE, as an adjunct to the PI-RADS scores, which can improve DRs of PCa in both AA and W men. Specifically, the combined use of *K*^*tran*^ and PI-RADS improved the detection of csPCa in TZ, for AA men decreasing the MRI-based racial diagnostic disparity significantly. We applied the single threshold value of qDCE to all lesions, regardless of the lesion location, for a simple strategy to update PI-RADS. However, a more refined approach can be considered by accounting for a specific lesion location in either TZ or PZ. The lesion-specific approach may further improve the csPCa detection as studies reported differences between TZ and PZ in the perfusion characteristics.^27^

We analyzed the diagnostic performance of mpMRI (DR and PPV) for total and csPCa lesions stratified by prostate zones in AA and W sub-cohorts. The DR for all lesions in the AA cohort was significantly lower than that in the W sub-cohort (57.1% vs. 66.7% respectively, p<0.001), and the PPV of csPCa in W men was significantly higher than that in AA men (77.1% vs. 72.7% respectively, p<0.001).

PI-RADS guidelines include different review criteria based on prostate zonal anatomy, and studies reported different diagnostic performances of mpMRI between TZ and PZ.^28,29^ We believe that investigating race-based information concerning prostate zonal anatomy will help clinicians to pay attention to specific areas with lower detection rates and positive predictive values. Furthermore, this may create future research that improves diagnostic performances of mpMRI by incorporating prior knowledge into learning-based methods.^30^

Our study has a few limitations. The first limitation is the relatively small cohort of AA men after matching commonly known clinical risk factors. Our initial study cohort comprised 885 patients who underwent mpMRI before prostatectomy from 2010 to 2020, and 4% of the study population were AA men, while the rest were mostly W men. Although this is reasonable because the racial composition of AA men in Greater Los Angeles is less than 10%, future studies can include performing a multi-institutional evaluation to bolster our sample sizes and improve its generalizability. The racial disparity between AA and W men in prostate cancer is considered to be caused by a combination of socioeconomic factors, environmental, and biological factors, we believe that the study is highly valuable as it was conducted in the second-largest metropolitan region in the United States by collecting the data for more than ten years. Secondly, a selection bias might be inherent to a surgical population, required for WMHP analysis. A few published articles showed the differences in MRI characteristics of PCa lesions and MRI diagnostic performance between AA and W men in biopsy populations. However, biopsy-confirmed histopathological findings commonly suffer from high uncertainty due to biopsy sampling error, interpretation variability, and lesions with borderline grades. Studies reported that more than 30% of the cases were upgraded, and more than 25% were downgraded, compared to WMHP.^31^ Therefore, despite potential selection bias, accurate matching of postoperative WMHP to MRI provides an accurate pathological assessment and image analysis. Also, it enables the analysis of false-negative lesions on MRI. Similarly, several studies have been conducted based on the surgical population when assessing the diagnostic performance of MRI.^32,33^ Lastly, the variability in qDCE measurement across different institutions and vendors remains a concern for the accurate and reproducible application of qDCE.^34^ A point-of-care perfusion phantom may reduce the inter/intra-scanner variability of qDCE measurement.^35,36^ Kim *et al*. recently demonstrated that the specificity of *K*^*trans*^ to detect csPCa was improved from 86% to 93% using a phantom-based error correction method.^37^ Regardless, significant differences in qDCE have been observed, and with increased reproducibility, the race-specific qDCE thresholds and the strategy of the updated PI-RADS could be improved for personalized patient care. Furthermore, our retrospective analysis led to including the surgical populations who equally received mpMRI and underwent radical prostatectomy between AA and W men. Other factors, such as timeliness of diagnosis, and extent of the disease due to differences in the use of MRI can be considered together with the quantitative MRI measures to understand and improve cancer care equity.^38^

## CONCLUSIONS

In this study, we attempted to detect differences in the diagnostic performance of mpMRI for detecting all and csPCa in AA and W sub-cohorts when assessed by prostate zones using WMHP correlation. We showed that quantitative MRI parameters in both pathology- and MRI-based csPCa lesions were significantly different between AA and W cohorts after matching the two groups by age, serum PSA levels, and prostate volume. This emphasizes that there are race-related differences in quantitative MRI measures that are not reflected in age, PSA, and prostate volume. The addition of qDCE to PI-RADS scores may reduce the racial disparity in the diagnosis of clinically significant prostate cancer by improving detection rates.

## Figures and Tables

**Table 1.** Patient characteristics before the propensity score matching.

	African American (N=37)	White men (N=74)	P-value
**Age years** **median (IQR)**	61(56–66)	63(58–68)	0.05
**PSA ng/ml** **median (IQR)**	6.7(5.6–8.2)	6.2(4.7–9.4)	0.4
**PSAD ng/ml/cc** **median (IQR)**	0.16(0.11–0.21)	0.16(0.12–0.24)	0.4
**PV cc** **median (IQR)**	41(33–55)	37(29–47)	**0.05**

**Table 2:** The detailed MRI sequence parameters.

	Spatial Resolution (mm^2^)	Slice Thickness (mm)	Matrix Size	Field-of-View (mm^2^)	TR/TE (ms)	b-values
**T2W** **MRI**	0.65 × 0.65	3.0	380 × 380	208 × 208	4000 / 109	-
**DWI**	1.6 × 1.6	3.6	160 × 94	260 × 216	3600 / 80	0, 100, 400, 800
**DCE-MRI**	1.6 × 1.6	3.5	160 × 160	260 × 260	4.2 / 1.5	-

**Table 3:** Diagnostic performance of mpMRI for detecting PCa lesions in African American and White men.

Diagnostic performance of mpMRI			African American men	White men	P-value
**ISUP grade ≥ 1 & PI-RADS ≥ 3**	**Number of lesions (TP/FN/FP)**		71 (36/27/8)	120 (74/37/9)	-
	**Cancer Prevalence (%)**	**Transition Zone**	31	29	0.6
		**Peripheral Zone**	61	63	
	Detection Rate* **(%)**	**All Pathology-based Lesions**	57	67	**< 0.001**
		**Transition Zone**	44	67	**< 0.001**
		**Peripheral Zone**	63	68	**< 0.001**
	PPV** **(%)**	**All MRI-based Lesions**	82	89	0.06
		**Transition Zone**	74	85	0.2
		**Peripheral Zone**	83	92	0.06

* Detection Rate = TP/(TP+FN)

** PPV = TP/(TP+FP)

**Table 4:** Patient and lesion characteristics after the propensity score matching.

		ALL	African American men	White men	*P-value*
**Number of patients**		111	37	74	-
**Age, years (IQR)**		61 (10)	61 (9.75)	62 (10)	0.7
**PSA, ng/ml (IQR)**		6.4 (3.9)	6.7 (2.5)	5.95 (4.6)	0.2
**PSAD, ng/ml/cc (IQR)**		0.14 (0.12)	0.15 (0.1)	0.13 (0.12)	0.5
**PV, cc (IQR)**		41 (23)	41 (23)	40 (22.5)	0.5
**Pathology-based Lesion**	**Number of lesions (lesions/patient)**	174 (1.57)	63 (1.7)	111 (1.5)	0.3
	**Solitary PCa lesions**	58	17	41	-
	**Multifocal PCa lesions**	116	46	70	-
	**ISUP grade 1**	50	22	28	-
	**ISUP grade2**	82	27	55	-
	**ISUP grade 3**	28	9	19	-
	**ISUP grade ≥ 4**	14	5	9	-
**MRI-based Lesion**	**Number of lesions (lesions/patient)**	124 (1.14)	41 (1.19)	83 (1.11)	0.7
	**PI-RADS 3**	29	11	18	-
	**PI-RADS ≥ 4**	97	33	64	-

All *p*-values were Mann-Whitney U test.

**Table 5:** Diagnostic performance of mpMRI for detecting csPCa lesions in African American and White men.

Diagnostic performance of mpMRI			African American men	White men	P-value
**ISUP grade ≥ 2 & PI-RADS ≥ 3**	**No. Lesions (TP/FN/FP)**		53 (32/9/12)	102 (64/19/19)	-
	**Cancer Prevalence (%)**	**Transition Zone**	26	25	0.8
		**Peripheral Zone**	67	68	
	**Detection Rate (%)**	**All Pathology-based Lesions**	78	77	**0.3**
		**Transition Zone**	67	80	0.03
		**Peripheral Zone**	81.2	76	> 0.9
	**PPV (%)**	**All MRI-based Lesions**	73	77	**< 0.001**
		**Transition Zone**	65	64	0.06
		**Peripheral Zone**	76	83	**< 0.001**

* Detection Rate = TP/(TP+FN)

** PPV = TP/(TP+FP)

**Table 6:** Quantitative mpMRI characteristics between African American and White men.

		K^trans^ (min^−1^ × 1,000)			k_ep_ (min^−1^ × 1,000)			ADC (10^−6^ mm^2^/s)		
		African American men	White men	P-value	African American men	White men	P-value	African American men	White men	P-value
**Pathology-based Lesions**	**ISUP grade=1**	207.0 ± 120.6	189.8 ± 149.9	0.7	613.7 ± 196.1	583.6 ± 268.2	0.7	953 ± 96.9	954 ±191	0.6
	**ISUP grade=2**	226.9 ± 77.7	186.3 ± 72.0	**0.02**	724.6 ± 182.4	634.8 ± 238.9	0.1	909 ± 211	899 ± 171	0.6
	**ISUP grade=3**	255.4 ± 170.4	166.2 ± 61.4	**0.05**	608.9 ± 189.6	685.3 ± 251.3	0.4	947 ± 273	899 ±165	> 0.9
	**ISUP grade≥1**	223.4 ± 108.8	188.2 ± 101.2	**0.03**	657.1 ± 192.0	641.2 ± 246.9	0.7	927 ± 204	888 ± 173	0.8
	**ISUP grade≥2**	232.2 ± 102.4	187.7 ± 79.7	**0.01**	680.4 ± 188.1	660.6 ± 237.8	0.6	909 ± 211	899 ± 171	0.6
	**ISUP grade≥3**	242.3 ± 141.5	190.3 ± 94.4	0.2	595.2 ± 174.7	711.4 ± 231.5	0.1	952 ± 229	848 ± 165	0.1
**MRI-based Lesions**	**PIRADS=3**	213.7 ± 94.6	194.5 ± 142.9	0.7	731.6 ± 200.7	632.5 ± 265.8	0.3	1155.0 ± 205.7	1009.2 ± 60.7	0.1
	**PIRADS≥3**	233.8 ± 107.5	186.5 ± 96.1	**0.01**	678.8 ± 188.7	654.8 ± 244.3	0.6	945.1 ± 222.1	901.5 ± 170.3	0.7
	**PIRADS≥4**	240.5 ± 112.0	184.2 ± 79.7	**0.01**	661.2 ± 184.3	661.1 ± 239.8	> 0.9	889.6 ± 193.2	868.1 ± 160.1	0.9

**Table 7:** Diagnostic performance of detecting ISUP grade ≥ 2 lesions using PI-RADS with qDCE thresholds (T_low_ and T_high_) tuned to different ethnic/race cohorts.

			AA men	W men	AA men	W men
			PI-RADS w/qDCE	PI-RADS ≥ 3	PI-RADS w/qDCE	PI-RADS w/qDCE
**ISUP grade ≥ 2**	Detection Rate* **(%)**	**All Pathology-based Lesions**	88	77	88	87
		**P-value**	0.2		0.8	
		**Transition Zone**	76	80	76	89
		**P-value**	0.2		**0.03**	
		**Peripheral Zone**	92	76	92	88
		**P-value**	**0.02**		0.4	
	PPV** **(%)**	**All MRI-base Lesions**	72	77	72	75
		**P-value**	**< 0.001**		**< 0.001**	
		**Transition Zone**	68	64	68	64
		**P-value**	0.1		0.3	
		**Peripheral Zone**	74	83	74	81
		**P-value**	**< 0.001**		**< 0.001**	

* Detection Rate = TP/(TP+FN)

** PPV = TP/(TP+FP)

## Data Availability

The datasets generated and analyzed during the current study are not publicly available but will be available from the corresponding author, K.S., on reasonable request, which will be reviewed and approved by the Institutional Data Sharing Committee.
